# The Role of *Lachnospiraceae* in Liver Diseases: Recent Advances and Clinical Application Prospects

**DOI:** 10.3390/ijms27167175

**Published:** 2026-08-11

**Authors:** Jincheng Feng, Xueling Wang, Huan Cao, Yu Zhang, Jianjun Xu, Guoliang Wang, Xiaodan Zhu, Shenghe Deng

**Affiliations:** 1 Center for Liver Transplantation, Union Hospital, Tongji Medical College, Huazhong University of Science and Technology, Wuhan 430022, China; fengjincheng25@163.com (J.F.);; 2 Department of Hepatobiliary Surgery, Union Hospital, Tongji Medical College, Huazhong University of Science and Technology, Wuhan 430022, China

**Keywords:** gut–liver axis, short-chain fatty acids, *Lachnospiraceae*, liver diseases, microbiome

## Abstract

Chronic liver diseases impose a considerable global public health burden, yet available treatment options remain largely inadequate. The gut microbiota exerts a key modulatory effect on liver disease pathogenesis via the gut-liver axis. Among them, *Lachnospiraceae*, a dominant bacterial family in the healthy adult gut, has drawn growing interest in recent years. *Lachnospiraceae* exert protective functions by producing short-chain fatty acids, participating in secondary bile acid conversion, and synthesizing active metabolites such as *N*-acetyl-glutamic acid, thereby maintaining intestinal barrier integrity and regulating host metabolic and immune homeostasis. Extensive evidence indicates that in cirrhosis, alcohol-associated liver disease, and metabolic dysfunction-associated steatotic liver disease, *Lachnospiraceae* abundance is consistently and significantly reduced, and this decrease is closely correlated with disease severity and adverse prognosis. In hepatocellular carcinoma, however, different members of *Lachnospiraceae* exhibit functional divergence, with some butyrate-producing genera decreasing while other subgroups may become enriched and influence the tumor immune microenvironment. Live biotherapeutic products based on *Lachnospiraceae* have achieved clinical breakthroughs in recurrent *Clostridioides difficile* infection and metabolic syndrome, providing important references for their translational application in liver diseases. This review systematically synthesizes the abundance changes and mechanisms of *Lachnospiraceae* across major liver diseases, evaluates their biomarker and therapeutic target potential, and seeks to provide fresh perspectives for precision microbiome-modulating strategies in chronic liver disease management.

## 1. Introduction

Chronic liver diseases impose a substantial global health burden, as underscored by recent epidemiological data [1,2]. Drawing upon the 2021 Global Burden of Disease database, the current worldwide prevalence stands at 1.7 billion cases, accompanied by 58.4 million incident cases and 1.4 million disease-related fatalities on an annual basis [3]. A closer look at temporal trends reveals a particularly concerning rise in MASLD among younger adults. Specifically, within the 15-to-49-year-old demographic, the age-standardized incidence has climbed at an average yearly rate of 0.72% over the three decades spanning 1990 to 2021 [3]. The toll exacted by cirrhosis is equally severe, claiming between 1.4 and 1.5 million lives per year and accounting for nearly 46 million disability-adjusted life years lost globally [4]. Notwithstanding advances in antiviral therapies and supportive care, current treatment options still fall short in multiple critical aspects. Strategies for preventing and managing advanced-stage cirrhosis and its major complications, namely hepatic encephalopathy (HE), ascites, and variceal hemorrhage, have yet to achieve optimal outcomes. Hepatocellular carcinoma (HCC) continues to present dual challenges, with suboptimal early diagnostic coverage and persistently high postoperative recurrence rates [5,6]. Together, these clinical gaps have directed research efforts toward previously underappreciated pathophysiological mechanisms and novel therapeutic approaches.

The gut–liver axis concept has provided a new framework for understanding the pathogenesis of liver diseases [7]. Anatomically and functionally, the gut and liver are intimately connected, with intestinal blood delivered directly into the liver via the portal venous system. This arrangement makes the liver the first organ to encounter gut derived substances, which include nutrients, microbial metabolites, and endotoxins [8,9]. Maintaining proper intestinal barrier function is critical for preserving the stability of the gut–liver axis [10]. When gut microbial dysbiosis leads to increased intestinal permeability, bacterial products such as lipopolysaccharides (LPS) can translocate through the portal circulation and reach the liver. Once there, these components activate Toll-like receptor signaling pathways, which in turn trigger a cascade of inflammatory responses and pathological changes within the hepatic tissue [10,11]. Considerable evidence has linked gut microbial imbalance to the pathogenesis and progression of multiple liver disorders, such as alcohol-associated liver disease (ALD), MASLD, cirrhosis, and HCC [12,13,14]. In cirrhotic patients, for instance, gut microbiome α-diversity is often markedly decreased, together with substantial shifts in microbial community composition. These alterations manifest as a decrease in commensal bacteria that produce short-chain fatty acids (SCFAs), alongside an overgrowth of opportunistic pathogens from the *Enterobacteriaceae* and *Streptococcaceae* families [15]. This dysbiosis and impaired intestinal barrier function operate in a mutually reinforcing manner, with both factors jointly accelerating the course of disease. In recent years, intervention strategies that target the gut microbiome and are built upon the gut–liver axis framework have attracted considerable attention in both basic science and clinical research settings. A range of therapeutic modalities, including synbiotics, probiotics, and fecal microbiota transplantation (FMT), have yielded encouraging outcomes in preclinical animal models as well as in early phase human trials. These approaches have been reported to enhance liver function, attenuate inflammatory responses, and lower complication rates [16,17]. Among these various approaches, *Lachnospiraceae* has been recognized for its protective effects against a broad spectrum of diseases.

*Lachnospiraceae* belonging to the class *Clostridia* within the phylum *Firmicutes*, is one of the most prevalent bacterial families in the healthy human intestinal tract [18]. Members of this family are obligate anaerobes and exhibit Gram-positive staining characteristics, serving a variety of protective functions in host physiological homeostasis. Their most recognized function is SCFA production, particularly butyrate, which fuels intestinal epithelial cells and supports intestinal barrier integrity [19]. In addition, *Lachnospiraceae* participates in the biotransformation of primary bile acids into their secondary forms and inhibits the overgrowth of intestinal pathogens through colonization resistance mechanisms [20]. It should be noted that changes in the abundance of different genera within *Lachnospiraceae* are not uniform across various liver disease contexts. This functional heterogeneity arises from genomic differences at the strain level, with certain members specializing in carbohydrate fermentation and butyrate synthesis while others are enriched with biosynthetic gene clusters that encode antimicrobial peptides [21]. Given their essential roles in metabolic and immune regulation, *Lachnospiraceae* has garnered growing interest in gut–liver axis research and liver disease studies. This review aims to provide a systematic synthesis of current knowledge on *Lachnospiraceae* in liver diseases, summarizing its disease-specific abundance patterns and mechanistic basis across cirrhosis, MASLD, ALD, and HCC, while critically assessing its potential as a diagnostic biomarker and probiotic therapeutic, with the goal of informing future research and clinical translation.

## 2. Biological Characteristics and Functions of *Lachnospiraceae*

### 2.1. Taxonomy and Ecological Niche in the Healthy Gut

*Lachnospiraceae*, a member of the class *Clostridia* (phylum *Firmicutes*), ranks among the most prevalent bacterial families in the mammalian gastrointestinal tract [19]. Metagenomic surveys have revealed that *Lachnospiraceae* and *Ruminococcaceae* together dominate the fecal microbiota of healthy adults, with *Lachnospiraceae* comprising on average 10–45% of total fecal bacteria [22]. Phylogenetically, *Lachnospiraceae* is closely related to *Ruminococcaceae* and *Eubacteriaceae* within the class *Clostridia*, and all three families contain butyrate-producing members; however, they exhibit notable differences [23,24]. Bacteria of the family *Lachnospiraceae* generally possess a broader substrate utilization spectrum, including various plant polysaccharides such as pectin and starch [25]. Phylogenomic studies have demonstrated that the adaptive evolution of this family to the digestive tract is accomplished through a combination of habitat-specific gene loss and horizontal gene transfer. Notably, butyrate-producing capacity is not a universal feature of *Lachnospiraceae*. Among the 30 *Lachnospiraceae* genomes analyzed, fewer than half carried genes related to butyrate synthesis, and the distribution of this function is partially attributable to horizontal gene transfer [19]. Taxonomically, *Lachnospiraceae* exhibits considerable diversity. To date, the NCBI taxonomy database includes 118 genera and 1941 species within *Lachnospiraceae*, and phylogenomic analyses suggest that its diversity remains underestimated [22,26]. At the functional level, different genera display distinct metabolic division of labor: *Roseburia*, *Coprococcus*, and *Anaerostipes* are major butyrate producers, closely associated with intestinal homeostasis and host psychological status [27,28,29,30]; some members of the type genus *Lachnospira* (e.g., *L. pectinoschiza*) are specialized in pectin degradation [31]; certain members of *Blautia* have garnered attention for their involvement in metabolic cross-feeding among microbial communities [32]; representative species within *Lachnoclostridium* (e.g., *L. scindens*) are renowned for their role in the 7α-dehydroxylation of bile acids [33]. Abundance changes in the genus *Dorea* have been associated with various disease states [34,35]. It is worth noting that some lineages within this family exhibit polyphyletic origins, and its taxonomic framework still requires further revision and refinement [23].

*Lachnospiraceae* begins to colonize the intestinal lumen shortly after birth, and its species richness and relative abundance continue to increase throughout the host’s lifetime, gradually establishing it as a stable core member of the gut microbial community [18]. In terms of spatial distribution, the colonization of *Lachnospiraceae* exhibits marked regional and species-specific patterns. For example, a detailed microbial mapping study along 13 anatomical sites of the mouse digestive tract revealed that at the distal cecum, the abundance of Lactobacillus drops sharply, whereas members of *Lachnospiraceae* increase significantly. However, studies on the human colonic luminal microbiota have revealed an opposite distribution trend, with viable bacterial abundance decreasing along the longitudinal axis of the intestine [36,37]. *Lachnospiraceae* engages in complex interactions with other gut microbial communities, primarily maintained through two mechanisms: metabolic cross-feeding and phage-mediated ecological regulation. Zhao et al. constructed a human gut bacteria–phage interaction network and found that communities dominated by *Lachnospiraceae* show linkages to temperate phages and metabolic synergy, forming a symbiotic and cooperative network; in contrast, communities dominated by *Bacteroidaceae* are associated with lytic phages and exhibit resource-competitive features. The study further confirmed that the metabolic coupling intensity of *Lachnospiraceae* is approximately 1.5 times that of *Bacteroidaceae*. Both network structures displayed marked disruption under conditions of inflammatory bowel disease and obesity, suggesting that the cooperation-dominated network led by *Lachnospiraceae* is critical for maintaining gut microecological stability [38]. Furthermore, a variety of endogenous and exogenous factors can significantly influence the colonization and abundance of *Lachnospiraceae*. Diet is one of the most important regulatory factors; dietary polysaccharides and fiber components can be efficiently fermented by *Lachnospiraceae* members to produce SCFAs, thereby promoting their growth. Bokulich et al. found that early-life antibiotic treatment leads to a marked depletion of *Clostridiales* bacteria, including *Lachnospiraceae*, thereby delaying the overall maturation of the gut microecosystem; cesarean section and formula feeding can also alter the early colonization patterns of *Lachnospiraceae* [39]. In summary, *Lachnospiraceae* occupies a highly specialized ecological niche in the healthy gut, and its metabolic interaction networks and responsiveness to external factors collectively determine its important role in the maintenance of host health.

### 2.2. Metabolic Functions

**Figure 1:**
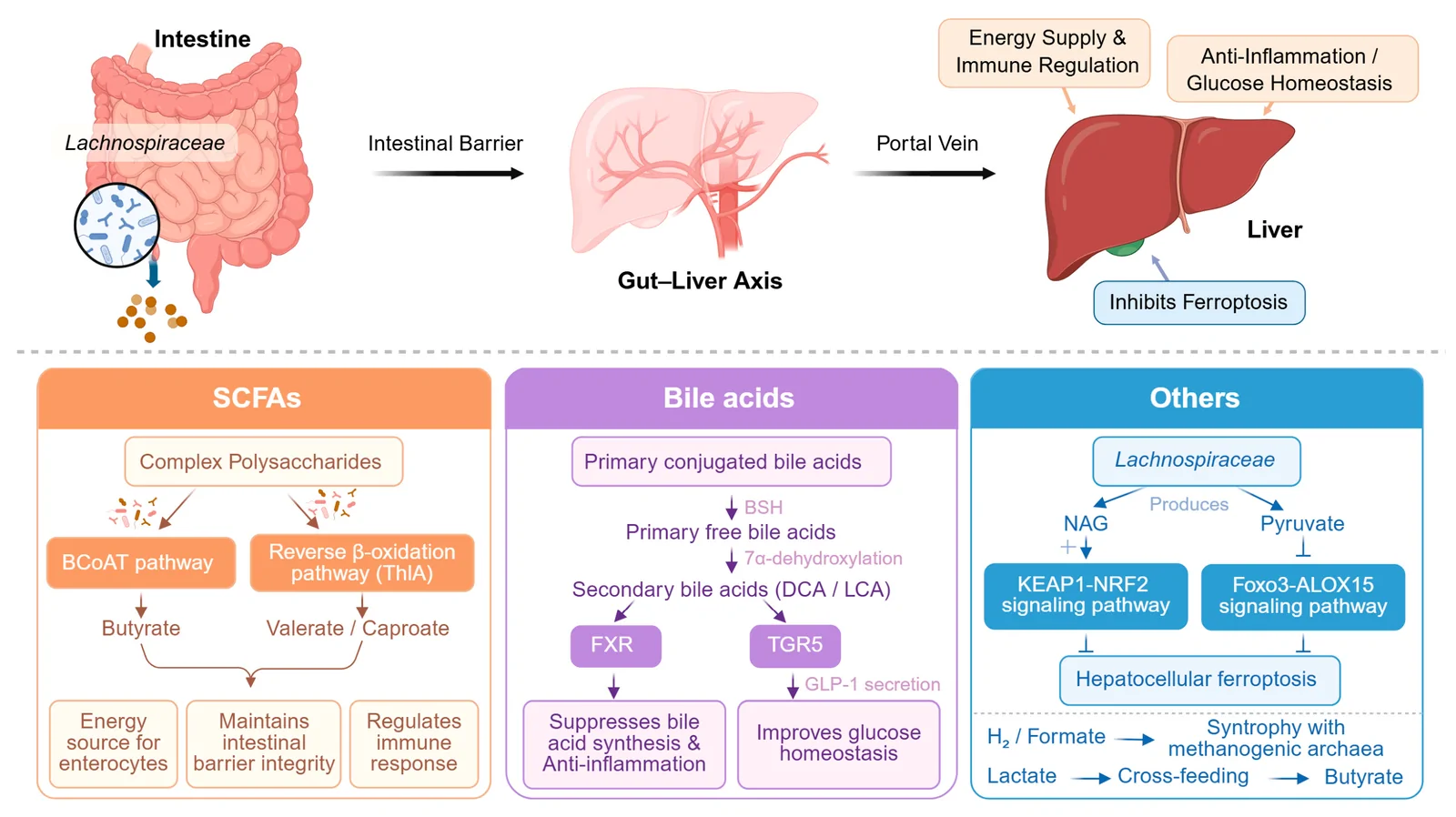
*Lachnospiraceae*-derived metabolites regulate liver diseases via the gut-liver axis. (**Upper panel**) Intestinal SCFAs, bile acids, and other metabolites translocate to the liver through the portal vein. (**Lower panel**) SCFAs production and effects (**left**); bile acid conversion to DCA/LCA activating FXR/TGR5 signaling (**middle**); NAG, pyruvate, H_2_, and lactate contributing to liver protection (**right**). Abbreviations: BSH, bile salt hydrolase; DCA, deoxycholic acid; LCA, lithocholic acid; FXR, farnesoid X receptor; TGR5, Takeda G protein-coupled receptor 5; NAG, *N*-acetylglucosamine.

As a prominent member of the human gut microbiome, *Lachnospiraceae* substantially influences host intestinal ecological balance and hepatic homeostasis through its metabolic output. This family harbors extensive and diverse metabolic capabilities, generating a range of bioactive metabolites that modulate hepatic physiology and pathology via the gut–liver axis [40,41] (Figure 1).

#### 2.2.1. SCFAs

*Lachnospiraceae* ranks among the principal gut commensals that produce SCFAs, notably butyrate [42]. Metagenomic analyses have demonstrated that SCFA synthesis-related gene clusters are broadly distributed across *Lachnospiraceae* genomes, equipping these bacteria to ferment dietary fiber and other complex polysaccharides into key metabolites including acetate, propionate, and butyrate [43]. These SCFAs not only serve as essential energy sources for intestinal epithelial cells but also function as critical signaling molecules in maintaining intestinal barrier integrity and modulating immune responses [20]. For butyrate production, *Lachnospiraceae* mainly utilizes the butyryl-CoA:acetate CoA-transferase (BCoAT) pathway. This pathway utilizes butyryl-CoA and acetate as substrates to directly generate butyrate via the catalytic action of BCoAT [44]. Studies have shown that the key genes of this pathway are widely distributed across different genera within *Lachnospiraceae*; for instance, *Coprococcus*, *Roseburia*, and *Anaerostipes* have all been confirmed to possess complete butyrate-synthesizing capacity [45,46]. Compared with other butyrate-producing bacteria such as *Faecalibacterium prausnitzii* (belonging to *Ruminococcaceae*), members of *Lachnospiraceae* exhibit heterogeneity in the genetic composition of butyrate metabolic pathways. For example, some *Lachnospiraceae* strains simultaneously retain genes of the butyrate kinase pathway, although their main flux still depends on the transferase pathway [44,46]. This metabolic difference may reflect functional specialization of distinct microbial groups within intestinal niches. In addition to butyrate, recent studies have uncovered novel functions of *Lachnospiraceae* in producing other SCFAs. Valerate and caproate have gained attention as gut metabolites in recent years. Research has found that specific members of *Lachnospiraceae* (e.g., *Anaerostipes hadrus* and *Coprococcus comes*) are capable of directly utilizing exogenous acetate, propionate, and butyrate as substrates for valerate and caproate synthesis through the reverse β-oxidation pathway. The key rate-limiting enzyme in this pathway is acetyl-CoA acetyltransferase (ThlA); differences in substrate preference among ThlA variants carried by different *Lachnospiraceae* strains (e.g., varying activity toward butyryl-CoA) determine whether the end product is valerate or caproate [41]. This finding significantly expands our understanding of the metabolic versatility of *Lachnospiraceae*.

#### 2.2.2. Bile Acid

*Lachnospiraceae* participates in the enterohepatic circulation of bile acids by enzymatically modifying host-derived primary bile acids. This process in turn affects both the compositional diversity and the signaling properties of the bile acid pool. Certain members of this family possess bile salt hydrolase (BSH) activity, with this enzyme mediating the deconjugation of conjugated bile acids such as glycocholic acid and taurocholic acid. Through this action, free bile acids are liberated [47]. Subsequently, specific *Lachnospiraceae* taxa are capable of catalyzing 7α-dehydroxylation reactions, thereby transforming free primary bile acids into secondary forms including DCA and LCA [48,49]. These microbially derived secondary bile acids function as essential signaling molecules that bind to host nuclear receptors and membrane receptors. Previous investigations have shown that secondary bile acids, including DCA and LCA, exert regulatory effects on FXR and TGR5. Specifically, DCA and LCA are potent agonists of TGR5; by activating TGR5, they stimulate GLP-1 release, consequently modulating energy metabolism and glucose balance. Meanwhile, secondary bile acids display distinct affinities for FXR when compared with primary bile acids. This differential binding collectively modulates the activity of the FXR signaling pathway, with downstream effects that include regulation of hepatic bile acid synthesis and modulation of inflammatory responses [50,51,52]. In pathological states like cirrhosis, *Lachnospiraceae* abundance declines markedly, resulting in diminished secondary bile acid synthesis and consequent weakening of FXR/TGR5-mediated protective signaling. This exacerbates intestinal barrier disruption and promotes endotoxemia [51].

#### 2.2.3. Other Metabolites

NAG is a recently reported hepatoprotective metabolite produced by *Lachnospiraceae*. Patients with ALD and corresponding animal models consistently exhibit diminished *Lachnospiraceae* abundance. Administration of *Lachnospiraceae* markedly raises NAG concentrations in both hepatic and circulating compartments. Mechanistically, NAG has been shown to activate KEAP1–NRF2 signaling in hepatocytes, thereby suppressing ferroptosis and attenuating alcohol-induced hepatic injury, inflammatory infiltration, and oxidative stress [40]. This finding not only uncovers the protective role of the *Lachnospiraceae*–NAG axis in ALD but also provides a theoretical basis for its development as a potential probiotic formulation. In addition to butyrate and the other SCFAs described above, carbohydrate fermentation by *Lachnospiraceae* also produces metabolites such as hydrogen and lactate. Certain *Lachnospiraceae* strains generate hydrogen and formate during carbohydrate fermentation, with yields regulated by substrate type and hydrogen partial pressure. During hydrogen production, *Lachnospiraceae* may form syntrophic relationships with methanogenic archaea, thereby influencing the overall balance of intestinal gas metabolism [53]. Meanwhile, lactate, as an intermediate metabolite, can be cross-fed between *Lachnospiraceae* and other microbial groups (e.g., *Ruminococcaceae*) for further conversion into SCFAs such as butyrate [54,55]. Our previous study also found that pyruvate, a central metabolic intermediate produced by *Lachnospiraceae*, attenuates ischemia–reperfusion injury in steatotic donor livers by inhibiting the Foxo3–ALOX15 signaling axis and suppressing hepatocyte ferroptosis [56]. Furthermore, *Lachnospiraceae* also contributes to amino acid fermentation and vitamin synthesis; the diverse organic acids and cofactors it produces collectively constitute a complex network of small-molecule metabolites that profoundly influence host liver health and disease progression [57].

## 3. Dysregulation and Functional Roles of *Lachnospiraceae* in Liver Diseases

Substantial evidence has shown that *Lachnospiraceae* abundance is frequently dysregulated in chronic liver diseases, though the impact direction varies by disease stage and bacterial genus. In cirrhosis, ALD, and MASLD, overall *Lachnospiraceae* abundance is markedly decreased, leading to impaired intestinal barrier function, augmented inflammation, and hepatocyte injury as a consequence of reduced production of protective metabolites including SCFAs and NAG, a pattern consistent with a protective role for this family. In HCC, however, the roles of different *Lachnospiraceae* members are divergent—some butyrate-producing genera are decreased, whereas certain subgroups may become enriched under high tumor burden conditions and may exhibit pro-tumorigenic features by influencing bile acid metabolism and the immune microenvironment (Table 1).

### 3.1. Lachnospiraceae and Cirrhosis

Cirrhosis represents the end-stage of diverse chronic liver diseases, and its pathogenesis is intimately linked to gut microbial imbalance and compromised intestinal barrier integrity. As a major SCFA-producing bacteria in the gut, *Lachnospiraceae* exhibits a consistently decreased abundance in patients with cirrhosis [81,82]. A meta-analysis pooling 53 studies with 5076 participants confirmed that cirrhotic patients had significantly lower gut levels of *Lachnospiraceae* and *Ruminococcaceae* than healthy controls. Conversely, opportunistic pathogens such as *Enterobacteriaceae*, *Pasteurellaceae*, and *Streptococcaceae* were substantially overrepresented in the cirrhosis group [83]. A separate 16S rRNA sequencing-based study similarly observed that fecal *Lachnospiraceae* proportion was markedly lower in patients with decompensated hepatitis B virus (HBV)-related cirrhosis than in healthy controls, and its abundance was significantly inversely correlated with Child-Pugh score, implying that this reduction parallels disease severity [84]. Furthermore, systematic reviews and meta-analyses have further indicated that the relative abundance of *Lachnospiraceae* and *Ruminococcaceae* in the HE group is substantially decreased relative to that in cirrhotic patients without HE, suggesting that *Lachnospiraceae* depletion correlates with cirrhosis itself and, moreover, with the development of its severe complication—HE [59]. In patients with alcoholic cirrhosis and alcohol use disorder (AUD), the restoration of SCFA-producing bacteria, including *Lachnospiraceae* and *Ruminococcaceae*, following FMT was significantly correlated with reduced alcohol craving and improved cognitive function, further supporting the protective role of *Lachnospiraceae* in the progression of cirrhosis [85].

The reduced abundance of *Lachnospiraceae* in cirrhosis arises from a complex interplay of factors, including gut microbial dysbiosis, bile acid dysregulation, epithelial barrier disruption, and persistent immune activation. Under cirrhotic conditions, *Lachnospiraceae* taxa equipped with 7α-dehydroxylating capacity, such as certain *Blautia* species, undergo substantial depletion. This loss interferes with primary-to-secondary bile acid conversion, resulting in a reduced secondary bile acid pool and consequently attenuating FXR- and TGR5-mediated control over hepatic bile acid synthesis, mucosal barrier integrity, and inflammatory responses [86,87]. Concurrently, the decreased concentration of bile acids entering the intestine selectively suppresses the proliferation of beneficial taxa like *Lachnospiraceae*, while creating conditions that favor the overgrowth of potentially pathogenic bacteria such as *Enterobacteriaceae* [87,88]. Breakdown of the intestinal barrier permits translocation of endotoxins such as LPS into the portal circulation, from which they gain access to the liver and trigger TLR4-dependent pro-inflammatory signaling cascades [89]. This hepatic inflammatory response in turn curtails hepatobiliary bile acid output, diminishing the supply of bile acids to the intestinal lumen and thus perpetuating a feed-forward loop of sustained dysbiosis and liver injury. *Lachnospiraceae* also serve as primary generators of SCFAs, particularly butyrate, within the intestinal ecosystem, and their contraction entails a commensurate drop in SCFA availability. Given that SCFAs provide colonocytes with an essential energy source while also governing tight junction expression and barrier function [90], their reduced production intensifies mucosal leakiness and fuels the ongoing inflammatory response. Among hospitalized cirrhotic patients, reduced counts of beneficial taxa including *Lachnospiraceae* independently correlated with the development of acute-on-chronic liver failure, multiorgan failure, and elevated 30-day mortality, implying that *Lachnospiraceae* abundance could hold value as a prognostic indicator in this population [91]. In a prospective cohort of cirrhotic individuals with concurrent sarcopenia and portal hypertension undergoing TIPS, those who achieved post-procedural reversal of muscle wasting displayed notable enrichment of SCFA-producing organisms, including *Faecalibacterium* and *Streptococcus*, alongside an upward trajectory of specific *Lachnospiraceae* lineages such as the ND3007 group. These observations raise the possibility that *Lachnospiraceae* modulate sarcopenia in cirrhosis via a gut–muscle signaling axis [60]. In our prior work, we identified that a *Lachnospiraceae*-derived metabolite, pyruvate, suppresses Foxo3–Alox15 axis activity, mitigates ferroptosis, and confers substantial protection against ischemia–reperfusion injury in both steatotic and non-steatotic donor livers during liver transplantation [56]. Overall, cirrhosis is marked by substantial *Lachnospiraceae* depletion, which drives the onset and evolution of liver disease and its associated complications via diverse mechanistic pathways, spanning bile acid dysregulation, SCFA deficiency, barrier dysfunction, and immune activation.

### 3.2. Alcohol-Associated Liver Disease (ALD)

ALD is driven by chronic excessive alcohol consumption and progresses through a continuum of disease stages. The gut microbiota plays a significant role in this pathological course via its effects on the gut–liver axis. A consistent body of clinical and experimental evidence indicates that *Lachnospiraceae* abundance is substantially diminished in both ALD patients and relevant animal models. Using 16S rDNA sequencing, Zhang et al. observed reduced *Lachnospiraceae* populations in the intestinal tract of ALD patients and ethanol-fed mice [40]. A pooled analysis of multiple 16S rRNA sequencing studies revealed significant depletion of several butyrate-producing families (*Lachnospiraceae*, *Ruminococcaceae*, and *Oscillospiraceae*) in AUD patients relative to healthy controls. Moreover, these taxa exhibited progressive depletion as AUD transitioned to ALD [92]. In a clinical trial comparing FMT against prednisone for severe alcoholic hepatitis, the FMT group showed better 90-day survival, with *Lachnospiraceae*, *Prevotella*, and *Veillonella* among the taxa associated with favorable outcomes [93]. The duration of alcohol exposure also appears to shape *Lachnospiraceae* composition, as the NK4A136 group showed enrichment after 4 weeks but not after 8 weeks of alcohol administration in animal studies [94]. From a genetic perspective, Mendelian randomization has provided evidence supporting a protective association of *Lachnospira* genus abundance against ALD [62]. In addition, dietary intervention with plant protein supplementation in ALD mice produced an 8.06-fold increase in *Lachnospiraceae* UCG 006, alongside alleviation of hepatic steatosis and fibrosis, suggesting that restoration of *Lachnospiraceae* parallels histological improvement [66].

*Lachnospiraceae* confers protection in ALD primarily through its metabolic products and its capacity to modulate host signaling pathways. Reduced *Lachnospiraceae* abundance coincides with pronounced suppression of butyrate synthesis; nonetheless, the butyrate precursor tributyrin effectively restores butyrate-producing bacterial populations and counteracts ethanol-induced hepatic steatosis, inflammation, and injury [61]. Clinical observations further substantiate this metabolic connection, showing that fecal SCFA levels in patients with alcoholic hepatitis correlate with reductions in *Lachnospiraceae* and *Ruminococcaceae* [95]. At the mechanistic level, *Lachnospiraceae* supplementation elevates NAG, activates the KEAP1–NRF2 pathway, and suppresses ferroptosis under alcohol exposure, thereby attenuating liver injury [40]. The effectiveness of various ALD intervention approaches hinges largely on their ability to restore *Lachnospiraceae* abundance, albeit through distinct downstream mechanisms. Several intervention studies have validated this relationship through distinct mechanistic routes. Polysaccharides derived from distiller’s grains confer hepatic protection by concurrently augmenting *Lachnospiraceae* abundance and activating the Nrf2/HO-1 axis [63]. Similarly, administration of *Lactobacillus paracasei* CCFM1120 reinstates butyrate-producing taxa including *Lachnospiraceae*, coincident with Nrf2/HO-1 pathway engagement and a marked improvement in hepatic antioxidant capacity [64]. A mixed fermentation extract of *Lonicerae japonicae flos* and *Cassiae semen* enriches *Lachnospiraceae* and *Blautia*, regulates lipid metabolism via the AMPK/ACC/SREBP1c signaling pathway, reduces serum alanine aminotransferase, aspartate aminotransferase, and triglyceride levels, and downregulates inflammatory mediators such as IL-1β, IL-6, and TNF-α [65]. The active polysaccharide ELP3, isolated from *Eucommia ulmoides* leaves, similarly drives *Lachnospiraceae* expansion while attenuating hepatic lipid deposition and inflammatory injury in ALD mice, an effect apparently mediated via the AMPK pathway and oxidative phosphorylation [96]. *Auricularia* polysaccharide AYP exerts protective effects against liver injury in ALD mice by enhancing *Lachnospiraceae* abundance and simultaneously modulating gut fungal community homeostasis [97]. Collectively, these findings indicate that restoration of *Lachnospiraceae* abundance represents a reliable indicator of therapeutic efficacy across diverse ALD interventions. Moreover, via its coordinated regulation of multiple metabolites and signaling pathways, *Lachnospiraceae* forms a multi-tiered protective network against alcohol-induced hepatic injury.

### 3.3. Metabolic Dysfunction-Associated Steatotic Liver Disease (MASLD)

MASLD is defined by hepatic steatosis together with peripheral insulin resistance, and its progression is intimately linked to intestinal microbial imbalance [67]. *Lachnospiraceae*, a predominant SCFA-producing family in the gut, has been the subject of extensive investigation in the context of MASLD. However, findings across studies remain notably heterogeneous, with considerable inconsistency regarding both the direction of abundance shifts and the specific genera involved. This heterogeneity may arise from differences in MASLD stages, variations in the racial and dietary backgrounds of study populations, discrepancies between fecal and mucosal microbiota sampling, and inconsistencies in sequencing platforms and bioinformatics pipelines [98,99,100]. Thus, due caution is required when interpreting available data in light of these confounding influences. In high-fat diet-fed MASLD mice, corosolic acid administration reshaped the gut microbiota, as verified by 16S rRNA sequencing and FMT experiments. Specifically, corosolic acid upregulated beneficial bacteria including the *Lachnospiraceae_NK4A136_group* while downregulating potentially harmful strains such as *Blautia*; mechanistically, the metabolite HAD-Car improves MASLD by inhibiting the cGAS–STING pathway [67]. Cinnamaldehyde similarly promotes this genus and exerts synergistic amelioration of MASLD via SIRT1/FOXO1-mediated autophagy enhancement [68]. Pyrodextrin-resistant starch enhances the growth of various *Lachnospiraceae* genera, including *Lachnospiraceae_NK4A136_group* and *unclassified_f__Lachnospiraceae*, and inhibits hepatic lipid synthesis and inflammatory responses by modulating bile acid metabolism [69]. Isorhamnetin increases the relative abundance of *Lachnospiraceae*, *Oscillospiraceae*, and *Ruminococcaceae*, alters the bile acid pool composition, activates the hepatic–ileal FXR signaling axis, accelerates enterohepatic circulation, and reduces dietary fat absorption [70]. In high-fat diet-fed mice, kefiran administration markedly reduced *Lachnoclostridium* abundance while promoting *Parabacteroides* and *Alistipes*, and ameliorated hepatic metabolic disorders through enhancement of one-carbon metabolism [101]. *Bacteroides ovatus* intervention enriches the *Lachnospiraceae_NK4A136_group* and reduces serum LPS and pro-inflammatory cytokine levels [102].

Population-based studies have provided additional insights into how *Lachnospiraceae* relates to clinical phenotypes of MASLD. Among non-diabetic NAFLD patients stratified by body mass index, lean individuals exhibited significant depletion of *Lachnospira* and *Subdoligranulum*, whereas *Escherichia–Shigella* was enriched; the combination of these genera effectively distinguished lean from non-lean NAFLD [73]. Data from the Rotterdam Study cohort linked *Lachnospiraceae* UCG-010 to MASLD risk [72]. In the context of disease progression, *Lachnospira* abundance was markedly lower in patients with MASLD-related fibrosis at stages F2–F4, pointing to a potential role for its reduction in fibrotic advancement [103]. Mendelian randomization studies further provided genetic evidence supporting the genus *Lachnoclostridium* as a protective factor for NAFLD [71]. In pediatric MASLD, baseline *Lachnospiraceae_ND3007_group* abundance discriminated between responders and non-responders to lifestyle interventions [74]. Mechanistically, Lingguizhugan Decoction upregulates *Lachnospiraceae_NK4A236_group*, inhibits the intestinal FXR–Fgf15 axis, and activates the hepatic CYP27A1/CYP7B1 alternative synthesis pathway [104]. Yellow tea polysaccharide studies have shown that *Lachnospiraceae_NK4A136_group* correlates significantly with alterations in specific bile acid species [105]. In a hepatocyte PLD1 knockout model, the abundance of the species-level *Lachnospiraceae* strain *Lachnospiraceae bacterium* 10-1 was substantially reduced, along with marked decreases in hepatic taurocholic acid and taurodeoxycholic acid levels, suggesting that PLD1 deficiency may modulate MASLD progression by altering gut microbiota–bile acid homeostasis [75]. Taken together, these observations suggest that distinct *Lachnospiraceae* members contribute to MASLD protection through diverse mechanisms, ranging from SCFA generation to bile acid metabolism; nonetheless, their specific roles appear to be genus-dependent and subject to variation across disease stages and intervention contexts.

### 3.4. Hepatocellular Carcinoma (HCC)

HCC is the most common primary liver cancer worldwide, typically arising from the stepwise progression of chronic liver diseases driven by multiple etiological factors, including HBV infection and MASLD [106]. The “gut–liver axis” concept has drawn growing interest in recent years, with the gut microbiota increasingly recognized as a pivotal modulator of host metabolic and immune homeostasis during HCC development [107]. Among these, the compositional changes in *Lachnospiraceae* exhibit complex stage-specific characteristics. Huang et al., in a study including 113 patients with HBV-related HCC, found that *Lachnospiracea incertae sedis* was significantly enriched in the feces of patients with non-small HCC (high tumor burden). Further integrated microbiome and host transcriptome analysis revealed that the abundance of this genus exhibited a significant negative correlation with the levels of immune-associated genes including *CD6* and *MAPK10* in liver tissues, both of which are protective genes associated with favorable prognosis, suggesting that enrichment of *Lachnospiracea incertae sedis* may promote tumor progression by suppressing antitumor immune responses. The same study further showed that metabolites of *Lachnospiracea incertae sedis* predominantly comprised bile acid species such as cholic acid and chenodeoxycholic acid, with elevated serum bile acid levels significantly correlating with unfavorable prognosis in HCC patients [76]. In a mouse model of metabolic dysfunction-associated steatohepatitis -related HCC, researchers similarly observed that the genus *Lachnospiraceae* UCG was significantly enriched in the gut of mice with a higher number of tumors [77]. Taken together, these observations imply that specific *Lachnospiraceae* subgroups may acquire pro-tumorigenic properties under high-tumor-burden conditions, potentially acting through modulation of bile acid metabolism and the immune microenvironment.

Conversely, several studies have consistently reported that butyrate-producing *Lachnospiraceae* members decline in abundance during HCC, coincident with progressive hepatic dysfunction. For instance, in elderly patients with HCC, the abundance of several genera within *Lachnospiraceae*, including *Blautia*, *Anaerostipes*, *Lachnospiraceae_ND3007_group*, and *Lachnospiraceae_FCS020_group*, were markedly lower than in healthy controls, with this decline correlating positively with serum transaminase levels [78]. This apparent paradox suggests that different members within *Lachnospiraceae* may play distinct roles at different stages or subtypes of HCC. In the immunotherapy setting, certain *Lachnospiraceae* members have been linked to clinical response. For example, *Lachnospiraceae bacterium* GAM79 was significantly enriched in hepatobiliary malignancy patients who achieved clinical benefit from anti-PD-1 immunotherapy; patients with higher abundance of this bacterium experienced significantly extended progression-free survival and overall survival, suggesting that this bacterium may improve patient prognosis by enhancing antitumor immune responses [79]. In addition, a review by Fukui systematically highlighted that in patients with cirrhosis and HCC, the ratio of beneficial autochthonous taxa represented by *Lachnospiraceae* and *Ruminococcaceae* to potentially pathogenic taxa such as *Enterobacteriaceae* is reduced, which is closely associated with early mortality and organ failure [108]. At the causal inference level, a Mendelian randomization study in the European population confirmed a significant positive causal association between increased abundance of the genus *Oscillospira* (a member of *Lachnospiraceae*) and elevated liver cancer risk [80]. In summary, the involvement of *Lachnospiraceae* in HCC transcends mere fluctuations in abundance; a deeper interrogation of this microbial signature holds promise for informing risk stratification, prognostic assessment, and the design of future microecology-based therapeutic interventions.

### 3.5. Effects of Standard Liver Disease Treatments on Lachnospiraceae Abundance

Standard liver disease treatments differ in their impact on *Lachnospiraceae* abundance, with antiviral regimens generally favoring its recovery. In the AAV-HBV mouse model, entecavir treatment reversed virus-induced gut dysbiosis, restored α-diversity, and increased the abundance of *Lachnospiraceae* [109].In hepatitis C virus (HCV) patients, the effects of direct-acting antivirals (DAAs) on *Lachnospiraceae* abundance varied depending on treatment response status and liver disease stage. Among those who achieved sustained virological response (SVR), gut microbial α-diversity approached healthy control levels, with Firmicutes emerging as the dominant phylum after treatment [110]. At the genus level, a review article reported that *Lachnospira* increased following DAA treatment in non-cirrhotic HCV patients [111]; in patients co-infected with HCV and human immunodeficiency virus (HIV), *Lachnospira* and *Subdoligranulum* significantly increased after SVR [112]. In addition, one study showed that *Blautia* exhibited the most pronounced enrichment among *Lachnospiraceae* following DAA therapy, and its recovery correlated with improvement in liver fibrosis [113]. However, in HCV-related cirrhotic patients, the increase in *Lachnospira* following DAA treatment was not statistically significant; the predominant changes observed were reductions in potentially pathogenic taxa, including *Enterobacteriaceae* and *Streptococcaceae* [114]. Following TIPS, changes in *Lachnospiraceae* showed a divergent pattern: patients who did not develop HE exhibited an increase in their autochthonous microbiota, with enrichment of *Lachnospiraceae* genera including *Coprococcus*, *Ruminococcus*, *Blautia*, and *Roseburia*, and these changes were negatively correlated with HE severity; conversely, patients who developed HE after TIPS showed a decrease in *Lachnospiraceae* abundance [58]. This observation is supported by another study in patients with HBV-related portal hypertension, which similarly found increased abundance of beneficial bacteria including *Coprococcus* and *Ruminococcaceae* in those who did not develop HE after TIPS [115]. Rifaximin appears to suppress specific members of *Lachnospiraceae*; among cirrhotic patients with refractory ascites, *Roseburia* abundance decreased substantially following rifaximin therapy [116]. For other commonly used agents such as non-steroidal anti-inflammatory drugs (NSAIDs) or statins; however, direct longitudinal data on *Lachnospiraceae* dynamics before and after treatment are currently limited, precluding firm conclusions at this time. Overall, conventional therapies exert heterogeneous effects on *Lachnospiraceae* abundance, underscoring the need to account for treatment-related confounders in future translational research. Whether these microbial shifts are causal contributors to, or simply correlates of, treatment outcomes awaits confirmation through well-designed prospective investigations.

## 4. The Therapeutic Potential of *Lachnospiraceae* as a Treatment Target

*Lachnospiraceae*, a core commensal bacterial family in the human gut, is increasingly recognized for its therapeutic potential across a range of diseases. In recent years, with growing appreciation of the intricate dialogue between the gut microbiome and host physiology, this bacterial family, once relegated to the simplistic label of “butyrate producers”, has progressively evolved from a mere microbial indicator toward the frontier of live biotherapeutic product development. Investigations into its therapeutic relevance in liver diseases, infectious disorders, metabolic disturbances, and autoimmune conditions are likewise progressing from preclinical validation toward clinical application (Table 2).

In an ALD mouse model, oral administration of *Lachnospiraceae* bacterium (ATCC BAA-2278) activated the KEAP1–NRF2 pathway through its metabolite NAG, subsequently curbing hepatocyte ferroptosis and preserving hepatic function [40]. In a randomized controlled FMT trial by Bajaj et al., infusion of donor stool enriched in *Lachnospiraceae* and *Ruminococcaceae* significantly reduced alcohol craving, improved cognitive performance, and decreased the incidence of serious adverse events in patients with alcohol-associated cirrhosis over a 6-month follow-up period [85]. In a Phase II HE trial, baseline *Lachnospiraceae* abundance in the recipient gut before transplantation was closely correlated with donor strain engraftment efficiency and the risk of HE recurrence [117]. In terms of genetic engineering, a modular genetic toolkit for *Lachnospiraceae* has been successfully developed, enabling controllable gene expression and protein secretion in the native commensal *Coprococcus comes*. Engineered *C. comes* capable of secreting IL-22 improved glucose homeostasis and attenuated hepatic steatosis in a mouse model of MASLD [118]. While the majority of these findings remain at the preclinical or early clinical stage, they collectively support the feasibility of *Lachnospiraceae*-targeted approaches for liver disease and offer valuable directions for future precision interventions grounded in the gut–liver axis.

Beyond liver diseases, the therapeutic promise of *Lachnospiraceae* has received more extensive validation across diverse pathological contexts. In rCDI, *Lachnospiraceae*-containing microbial consortia have reached pivotal milestones in clinical development. SER-109, an orally delivered microbiome agent sourced from donor feces and composed of purified Firmicutes spores, demonstrated a marked decrease in rCDI at the 8-week mark (12% versus 40% for placebo) in a Phase III randomized controlled trial [119]. This outcome underpinned its 2023 FDA approval as the first oral capsule live biotherapeutic for this indication, now marketed as Vowst™. VE303, an 8-strain consortium containing 5 *Lachnospiraceae* strains, similarly reduced rCDI recurrence rates in a Phase II trial and has completed Phase II proof-of-concept for HE [120]. In the metabolic field, a Phase I/II trial of *Anaerobutyricum soehngenii* (formerly *Eubacterium hallii*) in patients with metabolic syndrome demonstrated that fecal colonization abundance after 4 weeks of oral administration correlated positively with improvements in peripheral insulin sensitivity [121]; a trial also confirmed that duodenal delivery of this bacterium lowered glycemic variability, elevated postprandial GLP-1, and raised circulating secondary bile acids within 24 h [122]. In high-fat diet-fed mice, oral administration of *Blautia wexlerae* reduced body weight and multiple diabetic parameters, an effect linked to the anti-adipogenic and anti-inflammatory properties of its metabolites, including S-adenosylmethionine, acetylcholine, and L-ornithine [123]. Furthermore, in irritable bowel syndrome research, *Roseburia hominis* elevated fecal butyrate levels, relieved visceral hyperalgesia, and maintained intestinal occludin expression [124]. In a mouse model of herpes simplex virus type 1-induced Behçet’s disease, *Eubacterium rectale* ameliorated autoimmune symptoms by downregulating CD83 on dendritic cells and expanding NK1.1^+^ cell populations [125]. In a food allergy setting, introduction of *Anaerostipes caccae* into germ-free mice blocked β-lactoglobulin-induced allergic responses and lowered antigen-specific IgE concentrations [126]. A mixture of 17 bacterial strains, including 8 members of *Lachnospiraceae*, elevated the counts of regulatory T cells (CD4^+^FoxP3^+^) with anti-inflammatory function in the lamina propria of the colon across different mouse strains, and attenuated diarrhea and serum ovalbumin-specific IgE levels in an OVA-induced allergic diarrhea model [22]. In the field of tumor immunotherapy, *Eubacterium rectale* has been reported to boost the effectiveness of anti-PD-1 therapy in a mouse melanoma model, via a mechanism that involves L-serine utilization by this organism, which alleviates FOS/FOSL2-mediated NK cell suppression [127]. Together, these observations across diverse disease models substantiate the involvement of *Lachnospiraceae* in preserving intestinal barrier integrity, metabolic equilibrium, and immune homeostasis, while simultaneously providing a foundation for future mechanistic studies and clinical applications in hepatology.

## 5. Summary and Future Perspectives

In conclusion, *Lachnospiraceae*, a dominant bacterial family in the healthy adult gut, plays a critical role in maintaining intestinal barrier function and regulating host metabolic–immune homeostasis, effects achieved through SCFA generation, secondary bile acid conversion, and production of bioactive metabolites including NAG. In cirrhosis, ALD, and MASLD, Lachnospiraceae abundance is generally diminished, and this decline correlates with disease severity and complication risk [40,81]. However, in HCC, the roles of different *Lachnospiraceae* members are divergent—some butyrate-producing members are decreased, whereas certain subgroups may become enriched under high-tumor-burden conditions and influence the immune microenvironment. This functional heterogeneity suggests that caution should be exercised when targeting *Lachnospiraceae* as a therapeutic strategy [76,79]. These observations imply that shifts in *Lachnospiraceae* abundance represent more than a bystander effect of gut dysbiosis, but rather constitute a biologically relevant signal intimately tied to liver disease progression and clinical outcome. Its potential as a non-invasive biomarker for early warning and monitoring is therefore gaining attention. Although challenges such as functional heterogeneity and lack of prospective validation currently preclude its use as a standalone diagnostic tool, these limitations point toward clear optimization paths. With continued emphasis on butyrate-producing subgroups and individual strains, alongside integration of metagenomic analysis, longitudinal cohort data, and multi-marker approaches, *Lachnospiraceae* abundance may serve as a practical and dynamic adjunctive tool in clinical liver disease management.

The feasibility of targeting *Lachnospiraceae* as a biomarker or therapeutic candidate in liver disease has received initial support from recent studies, especially with clinical progress in FMT, engineered probiotics, and live biotherapeutic products (e.g., SER-109 and VE303) that collectively underpin translational efforts. However, this field still faces numerous challenges. First, substantial functional divergence exists not only among distinct genera but also across strains within the same family; however, the majority of current investigations remain confined to family- or genus-level resolution, hampering precise evaluation of strain-specific therapeutic potential. Second, available data derive largely from animal experiments and correlational analyses, with a conspicuous absence of large-scale, multicenter, prospective human interventional studies capable of establishing causality. Third, most members of *Lachnospiraceae* are strict anaerobes, and the preparation, preservation, and in vivo engraftment efficiency of live bacterial formulations pose industrial bottlenecks. Moreover, factors such as baseline microbiota composition, host genetic background, and dietary environment can significantly influence intervention outcomes, highlighting the need for developing precision therapeutic strategies based on microbiome stratification. Animal studies have suggested that host amylase loci may influence *Lachnospiraceae* abundance and associate with hepatic fibrosis phenotypes [128], yet direct evidence linking host genetic polymorphisms to *Lachnospiraceae* colonization or function in liver disease populations remains absent, warranting future cohort studies that integrate host genotyping with functional microbial analyses. Meanwhile, most studies have not adequately distinguished between the direct effects of drugs or therapeutic procedures on the gut microbiota and the secondary microbial changes resulting from improvements in liver function, inflammation, portal hemodynamics, nutritional status, or disease severity. This caveat merits special consideration in the interpretation of microbiome shifts associated with conventional interventions, including NSAIDs, statins, antiviral therapy, and TIPS. Finally, *Lachnospiraceae* may exert divergent or even opposing effects depending on the stage of liver disease, highlighting the need to define the optimal window for intervention.

Patient stratification based on microbiome profiling represents a critical step toward clinical implementation of *Lachnospiraceae*-targeted strategies. Different genera and strains within *Lachnospiraceae* exhibit functional divergence in liver disease, and this heterogeneity not only underscores the necessity for precise stratification but also provides a rich repertoire of targets for individualized intervention. *Lachnospiraceae* abundance correlates with disease severity, complication risk, and therapeutic response, while intervention efficacy tends to parallel the recovery of specific family members, a relationship that provides the biological rationale for its application as a stratification biomarker. To this end, we propose a multidimensional integrated stratification framework built around *Lachnospiraceae*, incorporating strain-level taxonomy, functional gene content, metabolite signatures, and host phenotypic traits to identify subgroups most likely to benefit, refine intervention timing, and minimize outcome heterogeneity. Future investigations should harness large-scale, multicenter prospective cohorts and multi-omic approaches to systematically characterize the temporal dynamics of key functional *Lachnospiraceae* strains over the course of liver disease and to establish their causal connections with clinical endpoints. Subsequent live-biotherapeutic intervention trials should incorporate the following design elements: prioritize representative strains with well-defined functions; establish rigorous manufacturing processes and dose-escalation protocols; enroll patients stratified by disease stage; adopt placebo-controlled, double-blind designs, with a fecal microbiota transplantation reference arm when feasible, and incorporate baseline microbiota typing as a stratification factor in randomization; evaluate endpoints that combine hard clinical outcomes with mechanistic biomarkers; conduct continuous sampling at baseline and at 3, 6, and 12 months post-treatment to assess strain colonization durability and microbial network recovery; and integrate host genetic background, dietary fiber intake, and medication history through multi-omics analysis to identify characteristics of high responders, thereby providing a basis for precision individualized intervention. Furthermore, the molecular regulatory networks of metabolites from different strains in hepatic inflammation, fibrosis, and the tumor immune microenvironment should be thoroughly investigated. Efforts should be actively directed toward the development of engineered live biotherapeutic products based on *Lachnospiraceae*, optimizing strain selection, delivery, and colonization strategies, and evaluating their safety and efficacy to accelerate clinical translation in MASLD, ALD, and HCC immunotherapy. Additionally, the interactions between *Lachnospiraceae* and other microbial communities, host genetics, and environmental factors should be emphasized, and multi-target combination regimens should be explored to achieve precision microbiome modulation in liver diseases. In summary, *Lachnospiraceae* functions as a key regulatory hub within the gut–liver axis, possessing considerable research importance and translational promise, with the potential to open new avenues for the prevention and management of chronic liver diseases.

## Figures and Tables

**Table 1:** Dysregulation and functional roles of *Lachnospiraceae* in liver diseases.

Disease Type	Genus/Species	Main Metabolites	Biological Effects
Cirrhosis	*Coprococcus*, *Blautia*, *Roseburia*	Butyrate	Abundance increased in patients without HE after TIPS, negatively correlated with HE severity [58]; *Roseburia* abundance significantly decreased in the intestinal mucosal microbiota of patients with HE [59]
	*Lachnospiraceae_ND3007_group*	SCFAs	Abundance increased after TIPS in cirrhotic patients with sarcopenia, consistent with the enrichment of SCFA-producing flora and the trend of sarcopenia reversal [60]
	*Lachnospiraceae bacterium*	Pyruvate	Alleviates ferroptosis by inhibiting the Foxo3-Alox15 signaling axis, ameliorates ischemia–reperfusion injury in liver transplantation for cirrhotic patients [56]
ALD	*Lachnospiraceae bacterium* (ATCC BAA-2278)	NAG	Activates the KEAP1-NRF2 pathway, inhibits hepatocyte ferroptosis, alleviates alcohol-induced liver injury [40]
	*Roseburia*	Butyrate	Butyrate precursor tributyrin restores butyrate-producing bacterial abundance and alleviates hepatic steatosis and inflammation [61]
	*Lachnospira*	SCFAs	Mendelian randomization supports a causal association between its increased abundance and reduced ALD risk [62]
	*Lachnospiraceae_NK4A136_group*	SCFAs	Upregulated by interventions such as Baijiu vinasse polysaccharides (activating Nrf2/HO-1 pathway) [63], *Lactobacillus paracasei* CCFM1120 (activating Nrf2/HO-1 pathway) [64], and honeysuckle-Cassia seed extract (regulating lipid metabolism via AMPK/ACC/SREBP1c pathway) [65], alleviating liver injury
	*Lachnospiraceae_UCG-006*	SCFAs	Abundance increased 8.06-fold after plant protein supplementation, accompanied by significant reduction in hepatic steatosis and fibrosis [66]
	*Blautia*	Butyrate	Honeysuckle-Cassia seed extract enriches this genus, regulates lipid metabolism via the AMPK/ACC/SREBP1c pathway and inhibits pro-inflammatory cytokine expression [65]
MASLD	*Lachnospiraceae_NK4A136_group*	SCFAs	Upregulated by multiple interventions including corosolic acid (inhibiting cGAS-STING pathway) [67], cinnamaldehyde (inducing autophagy via SIRT1/FOXO1) [68], P-RS (regulating bile acid metabolism) [69], and isorhamnetin (activating hepatic-ileal FXR signaling) [70]; improves insulin resistance, alleviates hepatic steatosis and inflammation
	*Lachnoclostridium*	Secondary bile acids	Mediates 7α-dehydroxylation; Mendelian randomization confirms as a protective factor for NAFLD [71]
	*Lachnospiraceae_UCG-010*	SCFAs, bile acid metabolites	Confirmed to be significantly associated with MASLD risk based on the Rotterdam Study cohort [72]
	*Lachnospira*	SCFAs	Significantly depleted in lean NAFLD patients; combined with *Escherichia-Shigella* and other genera effectively distinguishes lean from non-lean NAFLD [73]
	*Lachnospiraceae_ND3007_group*	SCFAs	Baseline abundance distinguishes responders from non-responders to lifestyle intervention in pediatric MASLD [74]
	*Lachnospiraceae bacterium 10-1*	Bile acid metabolites	Abundance decreased in the hepatocyte PLD1 knockout model, accompanied by decreased hepatic TCA and TDCA levels, involved in MASLD progression [75]
HCC	*Lachnospiracea incertae sedis*	Bile acids	Enriched in patients with high tumor burden; correlated with hepatic CD6, MAPK10 and other gene expression; may affect the tumor immune microenvironment via serum bile acids, associated with poor prognosis [76]
	*Lachnospiraceae_UCG*	Bile acid metabolites	Significantly enriched in the intestine of mice with higher tumor numbers in a mouse model [77]
	*Blautia*, *Anaerostipes*	Butyrate	Abundance significantly decreased in elderly HCC patients, associated with liver function impairment [78]
	*Lachnospiraceae_ND3007_group*, *Lachnospiraceae_FCS020_group*	SCFAs	Abundance significantly decreased in elderly HCC patients, associated with liver function impairment [78]
	*Lachnospiraceae bacterium-GAM79*	Not specified	Significantly enriched in hepatobiliary malignancy patients who benefited from anti-PD-1 immunotherapy, associated with prolonged PFS and overall survival [79]
	*Oscillospira*	Not specified	Mendelian randomization confirms a positive causal association between its increased abundance and elevated liver cancer risk [80]

Table footnote: NAFLD, non-alcoholic fatty liver disease; PLD1, phospholipase D1; TCA, taurocholic acid; TDCA, taurodeoxycholic acid; P-RS, pyrodextrin-resistant starch; TIPS, transjugular intrahepatic portosystemic shunt; PFS, progression-free survival. Genus and species names are italicized per microbiological nomenclature standards.

**Table 2:** The therapeutic potential of *Lachnospiraceae* as a treatment target.

Disease Area	Strain/Preparation	Main Effects	Potential Mechanism of Action	Study Stage
ALD	*Lachnospiraceae bacterium* ATCC BAA-2278	Improved liver function, attenuated liver injury [40]	Metabolite NAG activates the KEAP1-NRF2 pathway and inhibits hepatocyte ferroptosis	Animal study
Alcohol-associated cirrhosis	FMT	Reduces alcohol craving, improves cognitive function, reduces serious adverse events [85]	Restored SCFA-producing microbiota, repaired intestinal barrier, improved cognitive function and alcohol craving	Randomized controlled trial
HE	FMT	Baseline *Lachnospiraceae* abundance correlates with engraftment efficiency and recurrence risk [117]	Restored butyrate-producing beneficial bacteria, reduced inflammation and endotoxin levels, improved gut microecology	Phase II clinical trial
MASLD	Engineered *C. comes*	Improves glucose homeostasis, attenuates hepatic steatosis [118]	IL-22 regulates intestinal barrier and hepatic lipid metabolism, enhances mucosal immunity	Animal study
rCDI	SER-109 (Vowst™)	Reduces 8-week recurrence rate from 40% to 12%; FDA-approved [119]	Produces butyrate and other SCFAs, restores intestinal colonization resistance, inhibits *C. difficile* growth	Marketed (Phase III)
rCDI/HE	VE303 (containing 5 *Lachnospiraceae* strains)	Reduces rCDI recurrence rate; completed Phase II proof-of-concept for HE [120]	Restored gut microbial diversity, enhanced mucosal barrier and metabolic cross-feeding	Phase II clinical trial
Metabolic syndrome	*A. soehngenii*	Colonization associated with improved insulin sensitivity [121]; reduces glycemic variability, elevates GLP-1 and secondary bile acids [122]	Produces butyrate, modulates bile acid metabolism, promotes GLP-1 secretion	Phase I/II and Phase II clinical trials
Obesity/Type 2 diabetes mellitus	*Blautia wexlerae*	Reduces body weight and diabetic parameters [123]	Produces S-adenosylmethionine, acetylcholine, L-ornithine, etc., inhibits adipogenesis and inflammation	Animal study
Irritable bowel syndrome	*Roseburia hominis*	Increases butyrate, alleviates visceral hypersensitivity, protects intestinal barrier [124]	Produces butyrate, upregulates tight junction protein, modulates visceral sensory signaling pathways	Animal study
Behçet’s disease	*Eubacterium rectale*	Reduces CD83^+^ DCs, increases NK1.1^+^ cells, ameliorates symptoms [125]	Downregulates DC activation marker CD83, enhances NK cell responses, suppresses excessive immune responses	Animal study
Food allergy	*Anaerostipes caccae*	Prevents allergic reactions, reduces specific IgE [126]	Produces butyrate, promotes Treg differentiation, regulates Th1/Th2 balance, enhances oral tolerance	Animal study
Allergic diarrhea/Immunomodulation	17-strain consortium (including 8 *Lachnospiraceae* strains)	Increases Tregs, alleviates diarrhea, reduces IgE [22]	Induces regulatory T cell expansion, suppresses Th2-type inflammatory responses, maintains mucosal immune homeostasis	Animal study
Melanoma (immunotherapy)	*Eubacterium rectale*	Enhanced anti-PD-1 immunotherapy efficacy (prolonged survival, inhibited tumor growth) [127]	Depletes L-serine, relieves FOS/FOSL2-mediated suppression of NK cells, enhances antitumor immunity	Animal study

Table footnote: rCDI, recurrent *Clostridioides difficile* infection; DCs, dendritic cells; NK, natural killer; Tregs, regulatory T cells; IgE, immunoglobulin E; FDA, U.S. Food and Drug Administration. Bacterial genus and species names are italicized per microbiological nomenclature standards.

## Data Availability

No new data were created or analyzed in this study. Data sharing is not applicable to this article.

## Abbreviations

The following abbreviations are used in this manuscript:

ALD	alcohol-associated liver disease
AUD	alcohol use disorder
BCoAT	butyryl-CoA:acetate CoA-transferase
BSH	bile salt hydrolase
DCA	deoxycholic acid
DCs	dendritic cells
DAAs	direct-acting antivirals
FDA	U.S. Food and Drug Administration
FMT	fecal microbiota transplantation
FXR	farnesoid X receptor
GLP-1	glucagon-like peptide-1
HBV	hepatitis B virus
HCC	hepatocellular carcinoma
HCV	hepatitis C virus
HE	hepatic encephalopathy
HIV	human immunodeficiency virus
IgE	immunoglobulin E
LCA	lithocholic acid
LPA	lysophosphatidic acid
LPS	lipopolysaccharides
MASLD	metabolic dysfunction-associated steatotic liver disease
NAG	*N*-acetyl-glutamic acid
NAFLD	non-alcoholic fatty liver disease
NK	natural killer
NSAIDs	non-steroidal anti-inflammatory drugs
PFS	progression-free survival
PLD1	phospholipase D1
P-RS	pyrodextrin-resistant starch
rCDI	recurrent *Clostridioides difficile* infection
SCFAs	short-chain fatty acids
SVR	sustained virological response
TCA	taurocholic acid
TDCA	taurodeoxycholic acid
ThlA	acetyl-CoA acetyltransferase
TIPS	transjugular intrahepatic portosystemic shunt
TGR5	Takeda G protein-coupled receptor 5
Tregs	regulatory T cells
